# Organic nanofibers integrated by transfer technique in field-effect transistor devices

**DOI:** 10.1186/1556-276X-6-319

**Published:** 2011-04-08

**Authors:** Luciana Tavares, Jakob Kjelstrup-Hansen, Kasper Thilsing-Hansen, Horst-Günter Rubahn

**Affiliations:** 1 NanoSYD, Mads Clausen Institute, University of Southern Denmark, Alsion 2, DK-6400 Sønderborg, Denmark

## Abstract

The electrical properties of self-assembled organic crystalline nanofibers are studied by integrating these on field-effect transistor platforms using both top and bottom contact configurations. In the staggered geometries, where the nanofibers are sandwiched between the gate and the source-drain electrodes, a better electrical conduction is observed when compared to the coplanar geometry where the nanofibers are placed over the gate and the source-drain electrodes. Qualitatively different output characteristics were observed for top and bottom contact devices reflecting the significantly different contact resistances. Bottom contact devices are dominated by contact effects, while the top contact device characteristics are determined by the nanofiber bulk properties. It is found that the contact resistance is lower for crystalline nanofibers when compared to amorphous thin films. These results shed light on the charge injection and transport properties for such organic nanostructures and thus constitute a significant step forward toward a nanofiber-based light-emitting device.

## Background

In the last decade, much attention has been given to one-dimensional nanostructures due to their intriguing physics and in particular their application potential within for example electronics and optoelectronics [1-3]. *Inorganic *semiconducting crystalline nanowires made from, e.g., Si or III-V materials have been the focus of much research due to the ability to synthesize these in large numbers with well-defined properties, which has led to the demonstration of nanowire field-effect transistors [4,5], multicolor light sources [6], lasers [7], photo detectors [8,9], and solar cells [10,11].

Today, however, the interest in alternative materials to the more conventional inorganic semiconductors is increasing. One example is organic materials based on small molecules, which similarly can be self-assembled into crystalline nanostructures. This can be done either from solution [12,13] or by vapor deposition [14,15]. One of the main features of this class of material is its inherent tunability through chemical synthesis of the molecular building blocks [16], which enables the tailoring of the material properties for a specific application such as modification of the color of the luminescence output [17,18]. In addition, the optical and electrical properties [19] combined with low costs and fairly straight-forward processing (also on flexible substrates [12]) make these materials interesting candidates for nanoscale optoelectronic and photonic devices applications. The organic semiconductor para-hexaphenylene (*p*6P) can self-assemble into crystalline nanofibers structures that emit polarized, blue light upon UV excitation [20], and it has been shown to work as light-emitting material in organic light-emitting field-effect transistors (OLEFETs) [21].

A remaining challenge, however, is the integration of such organic nanofibers into the necessary surrounding circuitry such as metal electrodes for electrical biasing. Essentially, two different strategies can be used: (1) an in situ growth approach, in which the nanostructure is self-assembled directly on the device platform to establish the required electrical connections, and (2) a controlled transfer approach, in which pre-fabricated nanostructures are transferred to a device substrate for electrical wiring. Both strategies have been demonstrated on a wafer scale for inorganic nanowires [22,23], and we have recently demonstrated that the in situ growth approach is also possible for organic nanofibers [24], although with a nanofiber morphology that is inferior to epitaxially grown fibers. The transfer strategy is difficult to implement due to the fragility of the van-der-Waals-bond crystals. Previously, it was demonstrated how a few nanofibers could be transferred by a drop-casting technique and connected electrically to metal contacts for electrical two-point measurements, but this method was very time-consuming, with a low yield, and with loss of the parallel alignment [25].

In this study, we report results from a study of the electrical properties of *p*6P nanofibers implemented in different field-effect transistor (FET) configurations. The *p*6P nanofibers were first grown on a special growth substrate for epitaxial growth and then transferred to a silicon-based transistor platform. We have recently demonstrated in details how fast and large-scale transfer of organic nanofibers from the growth substrate onto a device platform enables an easy fabrication of a large number of devices (Tavares L, Kjelstrup-Hansen J, Rubahn H-G: Efficient Roll-on Transfer Technique for Well-Aligned Organic Nanofibers, submitted.) without damaging the morphology and optical properties of the fragile *p*6P nanofibers. Since the electrical characteristics of organic FETs are known to depend on the exact transistor geometry [26], we have studied three transistor geometries: bottom contact/bottom gate (BC/BG), bottom contact/top gate (BC/TG), and top contact/bottom gate (TC/BG) [26,27]. The BC/BG configuration is from a device fabrication point-of-view the easiest geometry, since no further processing is required after transfer of the organic material onto the device platform, while both the BC/TG and the TC/BG require additional deposition steps to form the top gate or the top contacts, respectively. However, the two latter geometries (known as the staggered configurations) usually exhibit superior device performance. This behavior is assumed to be due to the fact that the charges are injected not only from the edge of the electrodes (the case for a coplanar geometry) but also from the surface of the contacts [26].

## Results and discussion

**Figure 1:**
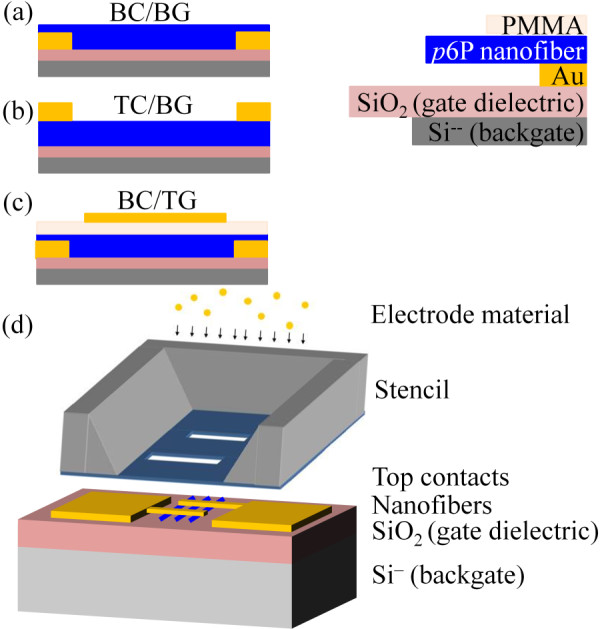
**The three different configurations used**: **(a) **BC/BG, **(b) **TC/BG, and **(c) **BC/TG. **(d) **Drawing of a device with TC/BG configuration prepared by deposition of the top contacts through a nanostencil.

The type 1 devices, which had a bottom contact/bottom gate (BC/BG, see Figure 1a) configuration, were ready for characterization directly after nanofiber transfer and annealing using the underlying highly doped silicon as the gate electrode. The type 2 devices had a top contact/bottom gate (TC/BG, see Figure 1b) configuration, and were prepared by depositing gold electrodes in high vacuum (range of 10^-6 ^mbar) on top of the transferred and annealed nanofibers through a nanostencil [28] with a pattern that gives top electrodes with the same dimensions as those used for the bottom contacts. In both bottom and top contact configurations, the contacts had dimensions of 10 μm × 200 μm, separated by a channel length of around 2 μm. Figure 1d shows an illustration of a TC/BG device with top contacts prepared by deposition through a stencil. The device type 3 was also a staggered configuration in a bottom contact/top gate (BC/TG, see Figure 1c) geometry.

**Figure 2:**
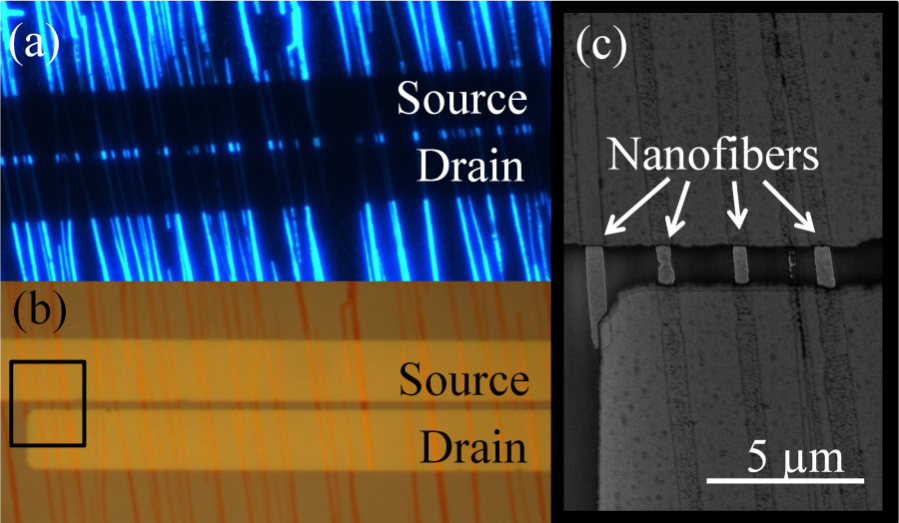
**Nanofibers in top contacts configuration**. **(a) **Fluorescence microscope image of nanofibers in the top contacts configuration. **(b) **White light microscope image of the sharp top contacts on nanofibers. **(c) **Scanning electron microscope image of the electrodes connecting to the nanofibers as indicated in (b).

Figure 2a,b,c show the nanofibers integrity and also the sharpness of the electrode edges on top of the nanofibers (TC configuration) (Figure 2b,c). The stencil used had 2 μm channel length but because of a blurring effect [29] during electrode deposition, a channel length of only approximately 1.5 μm is observed in the SEM image.

**Figure 3:**
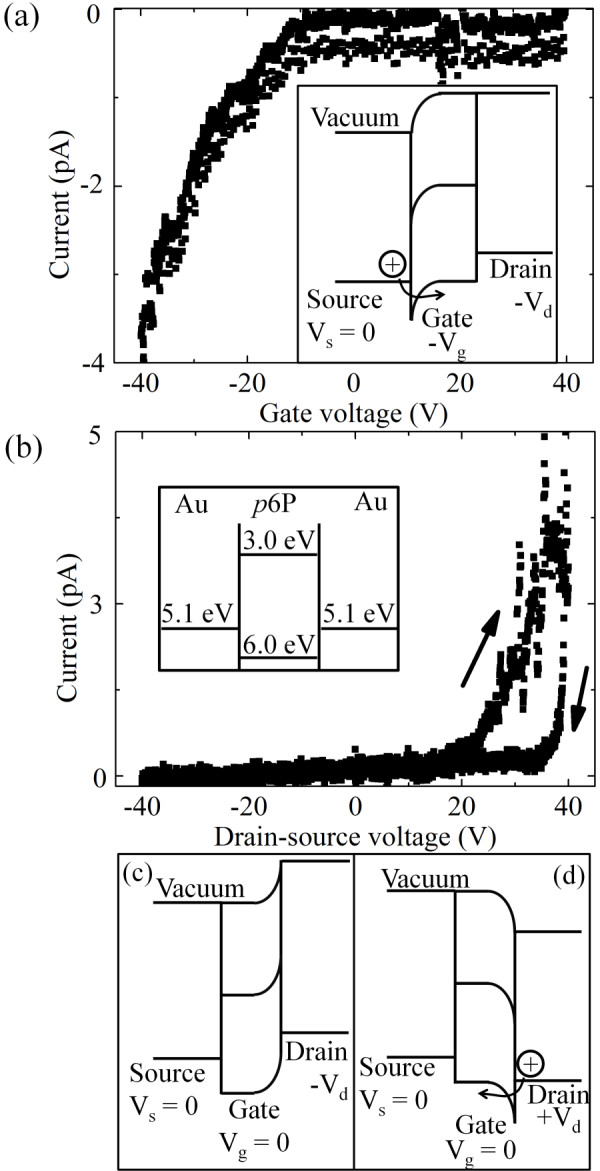
**Measured transistor characteristics for BC/BG nanofibers**. **(a) **Current versus gate voltage for *V*_ds _= -15 V. Inset shows schematic Mott-Schottky energy scheme for negative gate and drain voltages. **(b) **Current versus drain-source voltage for zero gate voltage. Arrows indicate the sweep direction. Inset shows energy level positions: the work function level for the gold drain and source electrodes (5.1 eV) and the LUMO (3.0 eV) and HOMO (6.0 eV) levels for *p*6P. **(c) **Mott-Schottky energy scheme for zero gate voltage and negative drain voltage. **(d) **Mott-Schottky energy scheme for zero gate voltage and positive drain voltage.

Figure 3a shows the measured transfer characteristics, i.e., current versus gate voltage for a drain-source voltage of -15 V for *p*6P nanofibers on a BC/BG device. The inset in Figure 3a is the Mott-Schottky energy scheme at negative gate and drain voltages which, however, do not account for interface traps states that could further reduce the current. The source-drain field allows only holes injected from the source electrode or electrons injected from the drain electrode to pass through the device and the measured characteristics clearly show that the transport is p-type, i.e., holes are injected from the source (see Figure 3a inset).

Figure 3b shows the current versus drain-source voltage for zero gate voltage for the same device. The inset schematically shows the energy level positions: the work function levels for the gold drain and source electrodes and the LUMO and HOMO levels for *p*6P. In Figure 3b, current flow is observed only for positive *V*_ds_. This must mean that the electrical characteristics are dominated by an injection barrier between the injecting metal electrode and the organic material. This is not unexpected given the energy levels shown in the inset that suggest an injection barrier for holes of around 0.9 eV. As shown in Figure 3d, a positive *V*_ds _then leads to downward band bending near the drain electrode and thereby a lowering of the hole injection barrier, while a negative *V*_ds _does not cause a similar band bending at the source electrode as would be required for hole injection in the opposite direction since the band bending again occurs at the drain electrode (see Figure 3c).

A hysteresis effect can also be observed in Figure 3b where the forward sweep is higher than the reverse sweep. This is assumed to be caused by trapping of the charge carriers [26,30,31]. We propose that the observed hysteresis is due to hole trapping close to the interface region between the injecting electrode and the organic material creating a space charge that reduces the band bending and thereby limits further hole injection, causing a lower back sweep current. We will elaborate on this aspect below.

**Figure 4:**
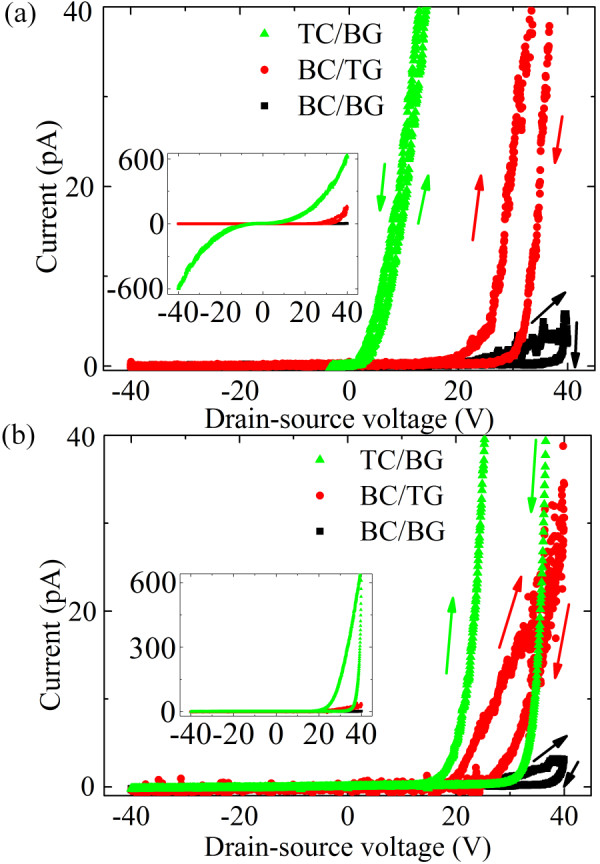
**Current versus drain-source voltage for zero gate voltage for (a) *p*6P nanofibers transferred from mica to a transistor platform and (b) *p*6P thin films for BC/BG, BC/TG and TC/BG configurations**.

Figure 4a shows current versus drain-source voltage for zero gate voltage for transferred *p*6P nanofibers for BC/BG, BC/TG, and TC/BG configurations, while the inset shows the same data plotted with a different current scale. Considering that approximately the same number of nanofibers was present in all the samples, the coplanar (BC/BG) configuration exhibits a lower output current than the staggered geometries due to a high contact resistance associated with the high injection barrier to the organic material [32]. In the staggered geometries (BC/TG and TC/BG), the charges are injected not only from the edge of the electrode but also from the surface of the contacts in the region where the source-drain electrodes overlap with the gate electrode and consequently charges are injected over a larger area leading to a lower contact resistance than in the coplanar (BC/BG) geometry [26].

The TC/BG configuration exhibits the highest output current. We propose that this is due to the smaller contact resistance between the nanofibers and the electrodes due to deposition of the electrodes under vacuum, which prevents water residues in the nanofiber-electrode interface in contrast to the bottom contact devices where the nanofiber-electrode interface is created under humid conditions during the transfer. As suggested by Bao and co-workers [33], moisture residing at the interface between the electrode and the organic material is expected to cause an increased contact resistance. Although our devices are annealed after fabrication, this can presumably not eliminate all water or water-transferred contaminants residing at the interface, since hysteresis is observed even after prolonged annealing. Also, metal penetrating into the organic material during electrode deposition can enable a better electrical contact [34,35].

The symmetric characteristics of the TC/BG device as opposed to the asymmetric behavior of the bottom connected devices can be observed in the inset of Figure 4a. Since no n-type behavior has been observed, this must mean that in the TC/BG devices the source electrode is injecting holes for negative drain-source voltages. The situation depicted in Figure 3c with band bending at the drain electrode is thus not valid for the top contact devices. Here, the main current limiting factor is the bulk nanofiber resistance giving rise to the observed symmetric output curve.

In Figure 4a, essentially no hysteresis is observed for the TC/BG configuration. Since these output characteristics are dominated by the nanofiber bulk as described previously, this suggests that the traps that cause the hysteresis must be spatially located near the injection region that governs the behavior of the BC devices.

Figure 4b shows the output characteristics for a 30 nm thick *p*6P film on similar transistor platforms. Around eight times more material was used to form the films compared to the material used to grow the nanofibers. The higher current density for the *p*6P nanofibers in comparison with the film must be consequence of the crystallinity of the nanofibers, i.e., *p*6P nanofibers have a long range order compared with thin films which is believed to favor a high charge-carrier mobility as a result of the π-conjugated coupling between the packed molecules [36] (see Figure 4a, b). The asymmetric curve observed for the thin film FET also in the TC/BG configuration in Figure 4b must be the result of a high contact resistance compared to the resistance of the film bulk. This implies that the contact resistance in TC devices is significantly lower for the crystalline nanofibers than for the amorphous film. In addition, the significant hysteresis observed for the injection limited thin film devices further support our conclusion of the traps being spatially located at the surface.

In Figure 4a,b, a drain current saturation is not observed. The channel length used was around 2 μm and the gate dielectric was 0.2 μm thick. It is well-known that if the channel length of a transistor is less than ten times the thickness of the gate dielectric, the space-charge-limited bulk current will be dominated by the lateral field due to the source-drain voltage preventing saturation since the gate voltage will not determine the charge distribution within the channel and consequently the "on" or "off" state of the transistor will not be observed [26].

**Figure 5:**
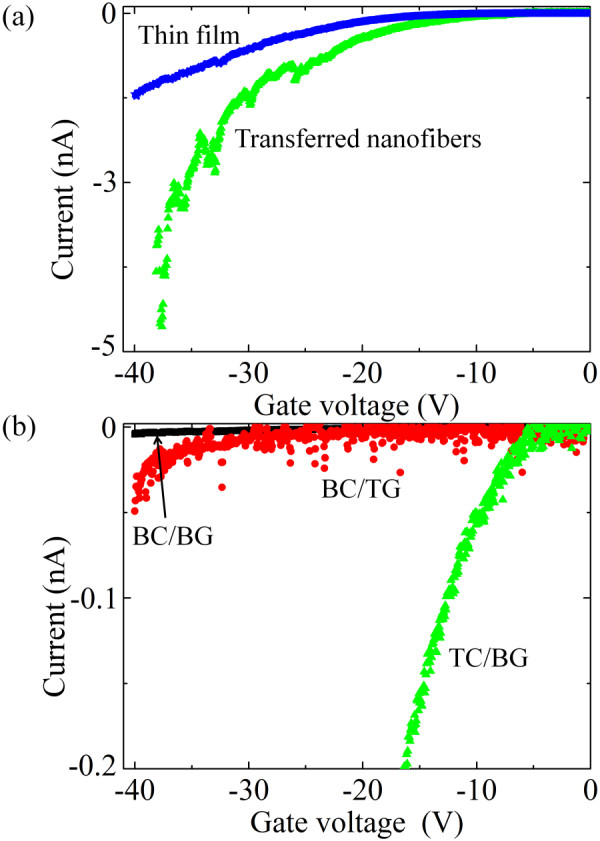
**Current versus gate voltage at *V*_ds _= -15 V for *p*6P (a) nanofibers and thin films in TC/BG configuration and (b) for nanofibers in BC/BG, BC/TG, and TC/BG configurations**.

Figure 5 shows the transfer characteristics, i.e., gate voltage sweep at a certain *V*_ds _for both *p*6P thin films and nanofibers. Figure 5a shows that the nanofibers conduct better than the thin films (as mentioned previously the film cross-sectional area is around eight times the nanofiber cross-section) and current saturation is not observed reinforcing the conclusion from Figure 4.

From Figure 5b, the subthreshold swings (*S *= d*V*_g_/d(log*I*_ds_)) [37] were obtained from the transfer characteristics of the *p*6P nanofibers on different transistor configurations to elaborate on the switching behavior.

From the data in Figure 5b, the subthreshold swing (*S*) for the nanofibers on BC/BG, BC/TG, and TC/BG configurations were found to be 13.7, 9.5, and 7.5 V/decade, respectively. The TC/BG configuration exhibits the lowest subthreshold swing being almost half that of the BC/BG device. For comparison, Klauk et al. [38] have studied the electrical characteristics for pentacene transistors with 100 nm SiO_2 _as the gate dielectric and found a subthreshold swing of only 0.7 V/decade. Our results is around a decade above this, however, this is not unexpected since the *p*6P mobility is significantly below that found in pentacene [21,38] and since our device geometry (here particularly the gate dielectric thickness) was not optimized for efficient switching.

## Conclusions

In this study, we have for the first time demonstrated integration of transferred organic nanofibers on different field-effect transistor platform configurations, which have been electrically characterized to reveal the significant differences in electrical performance between the different configurations. The coplanar device geometry has a high contact resistance and consequently a poor conduction compared to the staggered geometries. Within the staggered geometries, the top contact geometry shows superior performance to the bottom contact geometry presumably due to a cleaner interface between the contact and the organic material and due to metal penetration into the organic material during contact deposition. The better electrical connection of the top contacts results in the nanofiber transistor output characteristics being dominated by the nanofiber bulk as opposed to the bottom contact devices which exhibit injection limited behavior. A direct comparison of the crystalline *p*6P nanofibers with amorphous thin films shows that both materials exhibit p-type behavior but the fibers conduct significantly better owing to their better crystallinity.

Such electrical contacted organic nanostructures can have a range of applications, notably as nanoscale organic light emitters. These can be realized in similar field-effect transistor configurations and are therefore an obvious next subject to be studied. The performance of such organic transistors is influenced by a range of factors and optimization can therefore be pursued for example using other gate dielectrics [39], electrode materials [40], and by implementing nanofibers from other molecules [16].

## Methods

### Nanofiber growth

The nanofibers were prepared by vapor deposition of *p*6P molecules under high vacuum conditions (*p *< 10^-8 ^mbar) onto a heated muscovite mica substrate, which was cleaved in air before being immediately transferred to the vacuum chamber. During deposition (rate 0.1 Å · s^-1^), the substrate temperature was kept at 463 K. This enables the surface diffusion of the molecules and molecular clusters, which then agglomerate and form long, surface-bound, mutually parallel nanofibers with macroscopic lengths (up to millimeters), and nanoscopic cross sections (widths hundred to several hundred of nanometers and heights of several tens of nanometers) [15]. The herringbone stacked molecules in the fibers are oriented parallel to the substrate surface. The mean height and width of the nanofibers for 4 nm *p*6P deposition were around 40 and 250 nm, respectively, as determined by atomic force microscopy.

### Nanofiber transfer technique

The integration of the nanofibers onto the device platform took place via a special transfer technique, the details of which will be reported elsewhere (Tavares L, Kjelstrup-Hansen J, Rubahn H-G: Efficient Roll-on Transfer Technique for Well-Aligned Organic Nanofibers, submitted.). In short, the mica substrate with the nanofibers was fixed on the sidewall of a transparent cylinder with an appropriate diameter. The transparency of the cylinder helps to align the nanofibers to the device substrate and also to visualize when the mica and the device substrate are in contact to perform the transfer process. The device substrate was placed on a soft rubber platform to avoid compressing the nanofibers during the transfer, and the nanofibers were transferred by rolling the cylinder with the nanofibers onto the device substrates under conditions of high humidity. After transfer, the chips were annealed at 80°C for 20 min. This procedure was adopted to remove the water adsorbed during the transferring process.

### FET substrate preparation

Silicon-based device substrates were used for integrating the nanofibers with source, drain, and gate electrodes to form a field-effect transistor configuration. The substrates included elevated platforms that were used as receiver platforms for the nanofibers in the subsequent nanofiber transfer step. These platforms, which had a size of 1000 μm × 200 μm, were lithographically patterned on a highly doped silicon substrate with 200 nm thermally grown SiO_2 _and realized first by HF etching through the SiO_2 _layer followed by reactive ion etching 1 μm into the silicon to give a total platform height of 1.2 μm. On each receiver platform, two contact pads (390 μm × 180 μm) were prepared by photolithography, metal deposition (2 nm Ti/30 nm Au) and lift-off. We prepared two different types of substrates to be able to prepare both bottom contact (BC) and top contact (TC) devices. The TC device substrates were ready for nanofiber transfer after the preparation of the contact pads, while the BC substrates were processed additionally with one more sequence of photolithography, metal deposition (2 nm Ti/30 nm Au), and lift-off to form small, closely spaced electrodes, which were connected to the large contact pads, and onto which the nanofibers could be connected to span the gap. Gold was chosen as the electrode material due to its inertness and due to its high work function (5.1 eV) that promotes hole injection into the nanofibers.

The nanostencils were prepared from a 525 μm thick silicon wafer coated with a 100 nm low-stress silicon nitride (SiN) layer. The electrode pattern was realized in the frontside SiN layer by photolithography and reactive ion etching, and the membranes were released by photolithography and etching from the wafer backside in KOH solution (28 wt% KOH concentration at 80°C for approx. 9 h). A thin layer of photoresist was applied on the wafer frontside to protect the fragile membranes before dicing. After the initial tests of electrode deposition onto the nanofibers through the nanostencils, it was observed that the photoluminescence spectrum of the *p*6P nanofibers had changed and the nanofibers had a pronounced green appearance as opposed to the clear blue color of "perfect" nanofibers. We attribute this to the generation of defects in the nanofibers, which are known to give rise to peaks in the green part of the spectrum [41]. This could indicate that the thin SiN membrane shadow mask was too thin to protect the nanofibers against the radiation generated in the metal deposition (electron beam evaporation) system. The nanostencils were therefore coated with a thin metal layer to increase their ability to block the radiation that is expected to damage the nanofibers, and the nanofibers that were contacted using these improved nanostencils now exhibited the correct spectral appearance. The top gate on BC/TG geometry was prepared by applying 150 nm PMMA via spin-coating onto bottom contacted nanofibers to function as gate dielectric and applying a top gate electrode by gold deposition through a nanostencil with a suitable pattern (with dimensions of 120 μm × 320 μm) on the PMMA layer and on top of the electrodes. Tests were also performed to confirm the suitability of PMMA as gate dielectric by applying PMMA on a clean device substrate with BG/BC configuration. Here, no electrical conduction could be observed. Previous investigations have also shown that PMMA does not alter the *p*6P nanofibers' electrical characteristics and that the original *p*6P spectrum is also preserved after coating [41]. For the TC and the TG deposition, the alignment of the SiN stencil to the device substrate was done by hand under a white light microscope.

In addition to the nanofiber devices, *p*6P thin film [42] devices were also prepared for comparison of the electrical properties of crystalline nanofibers and amorphous thin films. The preparation method was identical with the exception of the nanofiber transfer step being replaced by vapor deposition of the *p*6P molecules directly onto the device substrates at room temperature resulting in a structure-less film.

### Characterization

The completed devices were inspected using white light microscopy, fluorescence microscopy (excitation wavelength of 365 nm), and scanning electron microscopy. The nanofiber dimensions were determined by tapping mode atomic force microscopy, and the field-effect transistor characteristics were recorded with a probe station and a labview-controlled characterization system based on a data acquisition card and voltage and current amplifiers.

## Abbreviations

BC/BG: bottom contact/bottom gate; BC/TG: bottom contact/top gate; FET: field-effect transistor; OLEFETs: organic light-emitting field-effect transistors; TC/BG: top contact/bottom gate.

## Competing interests

The authors declare that they have no competing interests.

## Authors' contributions

LT was involved in growing of the nanofibers and developing the FET substrates, made the transfer technique, transferred the nanofibers, performed the electrical measurements, contributed in the interpretation of data and drafted the manuscript. JKH developed the project, contributed developing the FET substrates, analyzing the data, drafting the manuscript and revised it, and have given final approval of the version to be published. KTH helped in growing of the nanofibers and developing the FET substrates. HGR revised it for important intellectual content.
